# Removal of Expression of Concern: Segneanu et al. *Helleborus purpurascens*—Amino Acid and Peptide Analysis Linked to the Chemical and Antiproliferative Properties of the Extracted Compounds. *Molecules* 2015, *20*, 22170–22187

**DOI:** 10.3390/molecules23010136

**Published:** 2018-01-10

**Authors:** Derek J. McPhee, Isabel C. F. R. Ferreira

**Affiliations:** 1 MDPI AG. Molecules Editorial Office, St. Alban-Anlage 66, 4052 Basel, Switzerland; 2 Mountain Research Centre, School of Agriculture, Polytechnic Institute of Bragança, Campus de Santa Apolónia, 1172, 5300-253 Bragança, Portugal

The article [1] published in *Molecules* was the subject of a law suit related to authorship. We previously published an Expression of Concern to highlight this fact to readers. A final judgment has now been made and no changes to the authorship are required.

We wish to reiterate that the published authors were found not to be at fault in choosing to publish the article in *Molecules* and were responsible for all aspects of the reported work.




